# Efficacy and safety of sitagliptin compared with sulfonylurea therapy in patients with type 2 diabetes showing inadequately controlled glycosylated hemoglobin with metformin monotherapy: A meta-analysis

**DOI:** 10.3892/etm.2015.2277

**Published:** 2015-02-09

**Authors:** LIQIONG HOU, TIEYUN ZHAO, YUNHUI LIU, YIYI ZHANG

**Affiliations:** Department of Endocrinology and Metabolism, West China Hospital of Sichuan University, Chengdu, Sichuan 610000, P.R. China

**Keywords:** diabetes mellitus, metformin, sitagliptin

## Abstract

Previous randomized controlled trials (RCTs) have reported conflicting results for the efficacy of sitagliptin and sulfonylurea therapy in patients with type 2 diabetes mellitus showing inadequate glycemic control with metformin monotherapy. To clarify these findings, a meta-analysis was conducted of the outcomes of all published RCTs comparing sitagliptin with sulfonylureas in the treatment of type 2 diabetes mellitus. Standard medical databases were searched to identify relevant English- and Chinese-language RCTs. RCT results were compared regarding the mean change in glycated hemoglobin (HbA1c) level; the proportion achieving <7% HbAlc; and a change in body weight. No significant differences were found between the metformin plus sitagliptin and metformin plus sulfonylurea groups regarding HbAlc or the proportion achieving <7% HbAlc, while the metformin plus sitagliptin group experienced fewer hypoglycemic events (P<0.00001) and a greater reduction in body weight (P<0.00001). Metformin plus sitagliptin therapy may decrease HbAlc values in patients with type 2 diabetes mellitus who are not achieving their glycemic targets with metformin monotherapy in a manner similar to metformin plus sulfonylurea therapy, whilst posing a lower risk of hypoglycemia, and yielding a more beneficial effect on body weight.

## Introduction

Type 2 diabetes mellitus has become a worldwide epidemic with a prevalence that has tripled in the last 30 years, and is predicted to affect >350 million individuals by 2025 (1). Despite lifestyle and pharmacological interventions, patients with type 2 diabetes mellitus continue to experience increases in glucose levels over time, which is likely to be as a consequence of declining β-cell function. One study found that approximately two-thirds of patients with type 2 diabetes mellitus in developed countries do not effectively control their glucose levels and that an even greater proportion does not do so in developing countries, particularly in China (2). A major reason for this failure is the progressive nature of type 2 diabetes mellitus, which makes it difficult for patients to maintain target levels of glycated hemoglobin (hemoglobin A1c; HbA1c) using traditional glucose-lowering agents, and usually requires them to take multiple antihyperglycemic agents (AHAs) to attain or maintain glycemic control.

Metformin, a commonly used oral antihyperglycemic agent used as a monotherapy and in combination with other antihyperglycemic agents, was introduced in the 1950s for the treatment of type 2 diabetes mellitus. Metformin has many advantages, including that it neither promotes weight gain nor causes hypoglycemia, it exerts beneficial effects on cardiovascular risk (3) and is well tolerated and inexpensive (4). Due to these advantages, clinical practice guidelines (5–8) recommend metformin as the first-line oral antihyperglycemic drug for treating most patients with type 2 diabetes mellitus when glycemic control cannot be achieved by lifestyle interventions alone. Sulfonylureas are frequently used as a second-line therapy if the use of metformin alone does not achieve acceptable glycemic control (9); however, an increased risk of hypoglycemia and weight gain can result from sulfonylurea treatment (10). Newer treatment options and combination therapies that sustain glycemic control with fewer such adverse effects are, therefore, being evaluated. Sitagliptin, a dipeptidyl peptidase-4 (DPP-4) inhibitor, is an incretin-based therapy that is effective and well tolerated when used in addition to metformin therapy (11,12). Furthermore, when added to metformin the risk of hypoglycemia with sitagliptin is similar to that observed using metformin with placebo (13). Several combination trials (14–19) have compared the efficacy and safety of sitagliptin with sulfonylurea therapy in patients with type 2 diabetes mellitus who are experiencing inadequate glycemic control (HbA1c >6.5 mmol/l and <10%) on metformin monotherapy; however, the trials reported conflicting results and used modest sample sizes (15–18). To clarify these findings, in the current study a meta-analysis was conducted of all the published RCTs to compare the efficacy and safety of combined metformin and sitagliptin therapy with combined metformin and sulfonylurea therapy in patients with type 2 diabetes mellitus who had been experiencing inadequate glycemic control when treated with metformin monotherapy.

## Materials and methods

### Literature search

The Medline, Embase, Cochrane Library, Chinese National Knowledge Infrastructure and Chinese Biomedical Literature databases were systematically searched to identify studies published in English between January 2000 and December 2012 or published in Chinese between January 1996 and December 2012 using the following search terms: Type 2 diabetes mellitus, type II diabetes mellitus, diabetes mellitus type 2, metformin, sitagliptin, sulfonylurea, glibenclamide, gliclazide, glipizide controlled-release tablets, gliquidone, glimepiride, dipeptidyl peptidase-4 and clinical trial. Following retrieval of the relevant articles, a manual search of the references was performed to identify the relevant trials. Attempts were also made to contact investigators for unpublished data and the full text of articles when deemed necessary for clarification or for more information. Two investigators reviewed all potentially relevant articles independently to determine whether they met all the inclusion criteria and none of the exclusion criteria.

### Study selection

Studies were included in the analysis if they met all the following inclusion criteria: i) Use of a prospective RCT; ii) comparison of combined metformin and sitagliptin therapy with combined metformin and sulfonylurea therapy in the treatment groups; iii) treatment of patients for ≥12 weeks; iv) inclusion of patients who had not been achieving their glycemic targets with metformin monotherapy; and iv) reporting of outcomes in terms of HbA1c values. Trials were excluded if they met one or both of the following exclusion criteria: i) Evaluation of the addition of more than one drug to metformin monotherapy and/or ii) inclusion of participants using background therapies other than metformin monotherapy. Methodological quality assessment was conducted using the Cochrane Collaboration’s tool for assessing the risk of bias in randomized trials. The RCTs were assessed for quality according to the criteria of i) method of randomization; ii) allocation concealment; iii) blinding of participants; iv) addressing of incomplete data; v) freedom of selective reporting; vi) comparability of groups at baseline; and vii) sample size calculation. The trials were independently reviewed and graded by two investigators who resolved any disagreements through discussion.

### Outcome measures

The primary outcome measure was the mean change in HbA1c values from baseline to study endpoint. Secondary outcomes included the proportion of participants achieving <7% HbA1c body weight, and the occurrence of hypoglycemia.

### Data extraction

Two investigators independently reviewed the titles, abstracts and full texts of articles for inclusion using standardized data extraction forms. Validity assessment was performed using the Jadad scale (20). Disagreements were discussed between investigators until agreement had been achieved. The following data were extracted from each trial: i) An individual reference identifier which indicated author and publication year; ii) fundamental study data, including indication, treatment duration, number of patients randomized, treatment aims and background medication; iii) patient characteristics at baseline, including mean age, gender, ethnicity, duration of type 2 diabetes mellitus, body mass index and HbA1c values; iv) quality measures, including the means of random sequence generation, allocation concealment, blinding and efficacy analysis; the dropout rate; and the funding source(s); and v) endpoint values, including the mean change in HbA1c values; the number of patients achieving <7% HbA1c; the change in body weight and the incidence of hypoglycemia.

### Statistical analysis

The analyses were performed using Review Manager (version 5.0; The Cochrane Collaboration, Copenhagen, Denmark) and Stata (version 10; Stata Corp, College Station, TX, USA) software. The heterogeneity of treatment effects among the studies was formally tested with Cochrane’s test at a significance level of P<0.1 and determination of the I^2^ statistic, with I^2^>50% considered an indication of significant heterogeneity between two trials. A random-effects model was used in the presence of heterogeneity and a fixed-effects model in the absence of heterogeneity. The weighted mean difference (WMD) or odds ratio and its 95% confidence interval (CI) for each outcome relative to the control were calculated for continuous and dichotomous variables, respectively. Studies were excluded from the meta-analysis if insufficient information was provided to enable standard error calculation.

## Results

### Study selection

**Figure 1:**
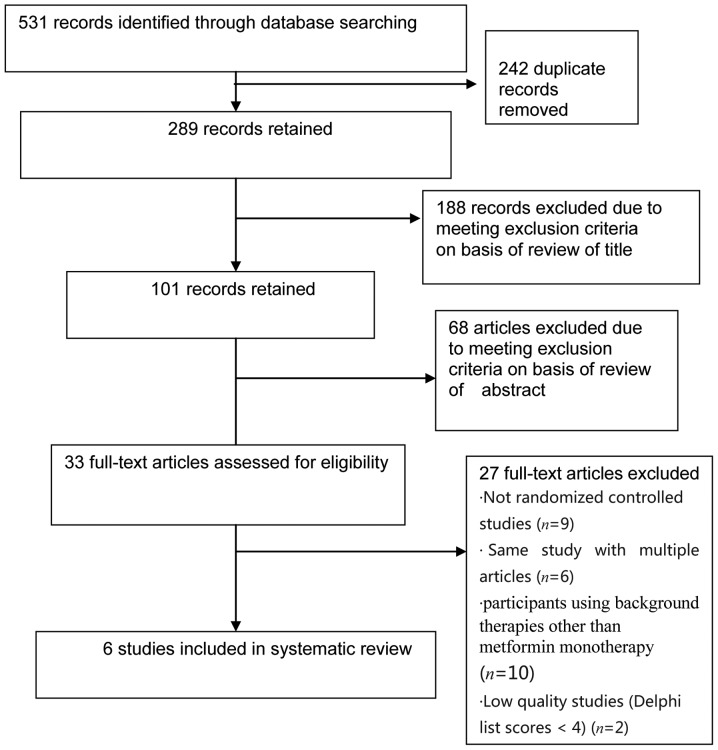
Flow chart of search results.

The database search results are summarized in Fig. 1. Among the 33 full-text articles that were assessed for eligibility, six reported the results of six RCTs (14–17,19,20) that fulfilled all the inclusion criteria and none of the exclusion criteria. The main reasons for the exclusion of six potential RCTs were the evaluation of the addition of more than one drug with metformin monotherapy, the inclusion of participants undergoing background therapies other than metformin, trial durations of <12 weeks, and a lack of participant randomization.

### Study and patient characteristics

Table I summarizes the characteristics of the six RCTs and the 3,585 participants that they included. As can be observed, the mean patient age ranged from 53 to 59 years, the percentage of male patients from 48.3 to 62.9%, and the baseline HbA1c level from 7.3 to 8.8%.

### Methodological quality and risk of bias

Table II shows the results of the assessment of risk of bias. As can be observed, the studies were found to be of moderate to high quality, with the majority fulfilling five to seven of the seven quality criteria. Specifically, one study fulfilled three criteria, one study fulfilled five criteria and the remaining three studies fulfilled seven criteria. The use of blinding was not deemed practical in several RCTs.

### Change in HbA1c values and HbA1c goal

**Figure 2:**
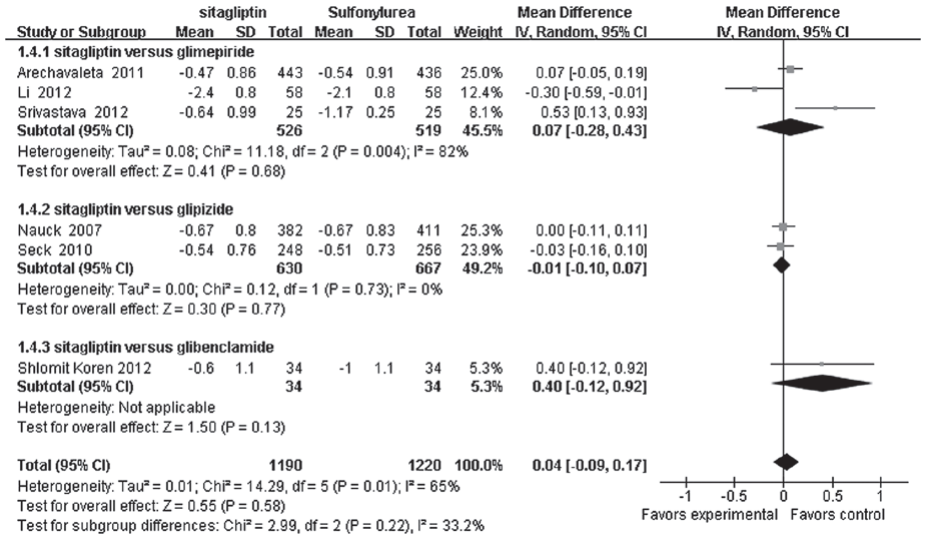
Comparison of changes in glycated hemoglobin between the metformin plus sitagliptin and the metformin plus sulfonylurea groups. SD, standard deviation; CI, confidence interval; df, degrees of freedom.

**Figure 3:**
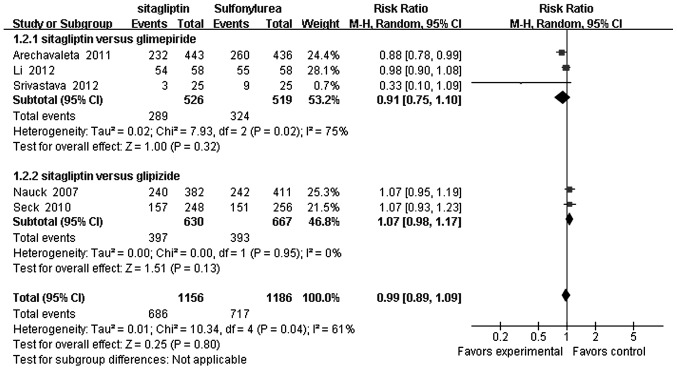
Comparison of achievements of <7% glycated hemoglobin between the metformin plus sitagliptin and the metformin plus sulfonylurea groups. M-H, Mantel-Haenszel; CI, confidence interval.

Fig. 2 shows the results of the meta-analysis of the change in HbA1c from baseline (i.e., the effect estimate), the primary outcome of the six RCTs that reported a change in HbA1c for 2,410 subjects. As can be observed, no significant differences were found between the metformin plus sitagliptin and the metformin plus sulfonylurea groups (WMD=0.04, 95% CI −0.09 to 0.17, P=0.58). Fig. 3 shows the results of the meta-analysis of the risk ratio for achieving <7% HbA1c for the five RCTs that reported the achievement of this goal for 2,342 subjects. As can be observed, no significant differences were found between the metformin plus sitagliptin and the metformin plus sulfonylurea groups in the attainment of this goal [risk ratio (RR)=0.99, 95% CI 0.89–1.09, P=0.80].

### Body weight

**Figure 4:**
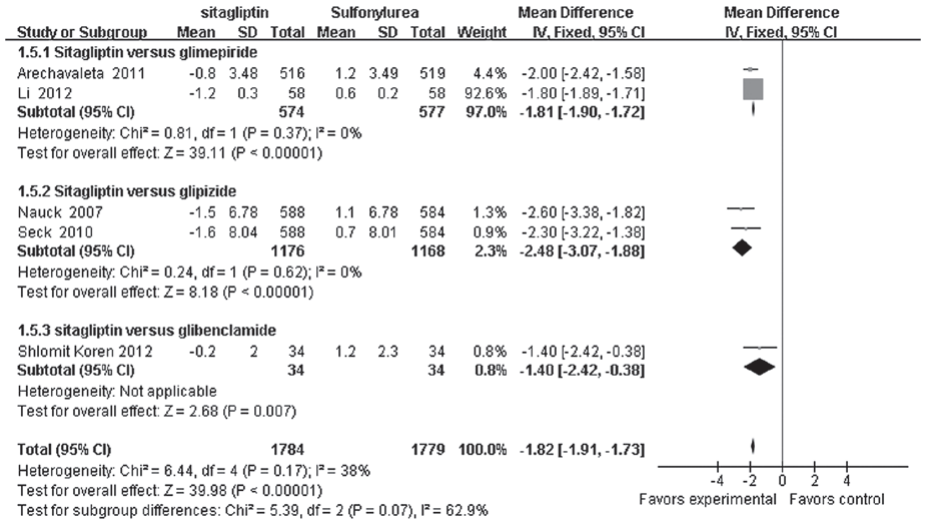
Comparison of changes in body weight between the metformin plus sitagliptin and the metformin plus sulfonylurea groups. SD, standard deviation; CI, confidence interval.

Fig. 4 shows the results of the meta-analysis of the change in body weight for the five trials that reported a change for 3,563 subjects. As can be observed, the metformin plus sitagliptin group was found to experience a significantly greater loss in body weight compared with the metformin plus sulfonylurea group (WMD=−1.82; 95% CI, −1.91 to −1.73; P<0.00001).

### Hypoglycemic events

**Figure 5:**
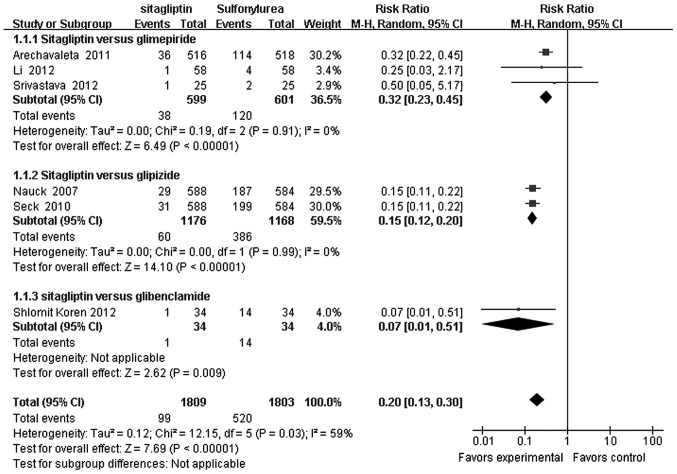
Comparison of occurrence of hypoglycemic events between the metformin plus sitagliptin and the metformin plus sulfonylurea groups. M-H, Mantel-Haenszel; CI, confidence interval.

Fig. 5 shows the results of the meta-analysis of the six RCTs that reported that 3,612 patients had experienced at least one hypoglycemic event. As can be observed, the metformin plus sitagliptin group was found to experience significantly fewer hypoglycemic events compared with the metformin plus sulfonylurea group (RR=0.20; 95% CI, 0.13–0.30; P<0.00001).

## Discussion

The results of the meta-analyses conducted in this study strongly indicate that the addition of sitagliptin therapy to the regimen of patients with type 2 diabetes mellitus who are currently undergoing metformin monotherapy but failing to achieve their glycemic targets can result in a reduction in HbA1c values similar to that resulting from the addition of sulfonylurea therapy to metformin monotherapy. Metformin plus sulfonylurea therapy, however, was not found to lower the risk of hypoglycemia to the same extent as metformin plus sitagliptin therapy. Since sulfonylurea stimulation of insulin secretion is not strictly glucose dependent (21), sulfonylurea agents continue to stimulate insulin secretion even with falling glucose concentrations (22). By contrast, sitagliptin inhibits the enzymatic degradation and inactivation of glucagon-like peptide-1 (GLP-1), thus increasing endogenous GLP-1 and gastric inhibitory polypeptide levels (23,24). GLP-1 then potentially stimulates insulin secretion and inhibits glucagon release effects that disappear when glucose levels approach normal concentrations (25). Sitagliptin also induces stimulation of insulin release and the suppression of glucagon release in a glucose-dependent fashion.

The results also suggest that metformin plus sitagliptin therapy results in greater weight loss compared with metformin plus sulfonylurea therapy. Regarding the underlying mechanism, sitagliptin can increase endogenous GLP-1, which, by delaying gastric-emptying, increases satiety, resulting in significant weight loss (26). This beneficial effect is important, as weight gain is a common side effect of sulfonylurea treatment that may be related to a sulfonylurea-induced increase in insulin concentrations (27).

The present study had two primary strengths and three major limitations that should be considered when reviewing the results. Regarding its strengths, the study authors ensured that only high-quality evidence was examined by limiting the inclusion of studies to only double-blind RCTs. Furthermore, as the first systematic review, to the best of our knowledge, to compare the efficacy and safety of sitagliptin and sulfonylurea therapies in the treatment of patients with type 2 diabetes mellitus experiencing inadequate glycemic control with metformin monotherapy, this study filled an important research gap. Regarding its limitations, the study examined RCTs that were conducted for varying lengths of time (range, 12–104 weeks), which may affect the extent to which the results can be compared. In addition, as the RCTs did not report economic indicators, the present study was not able conduct the relevant economic analysis. Such analysis is important, as cost is a significant consideration in therapeutic decision making in order to support the allocation of sufficient healthcare resources for the treatment of type 2 diabetes mellitus and its complications, particularly in light of its increasing global prevalence. Finally, as none of the RCTs were designed to compare the cardiovascular endpoints of the two study groups, any conclusions regarding outcomes, such as cardiovascular morbidity or mortality, should be considered with caution and only be accepted following confirmation by additional investigation by more RCTs.

In conclusion, the addition of sitagliptin therapy to the regimen of patients with type 2 diabetes mellitus who are not achieving their glycemic targets with metformin monotherapy may result in a reduction in HbA1c values in a manner similar to the addition of sulfonylurea therapy to metformin monotherapy while posing a lower risk of hypoglycemia and resulting in a greater loss of weight.

## Figures and Tables

**Table I:** Characteristics of studies and patients in six randomized controlled trials.

First author, year, country (ref)	Interventions	Patient characteristics	Duration (weeks)	Outcomes measured
Seck, 2010, USA (17)	Sitagliptin 100 mg qd + metformin ≥1500 mg qd Glipizide 5–20 mg qd + metformin ≥1500 mg qd	N=1172 Sitagliptin + metformin, n=588; glipizide + metformin, n=584 Mean age (years): Sitagliptin + metformin, 57.6; glipizide + metformin, 57.0 Sex ratio (%): Sitagliptin + metformin, 57.3M/42.7 F glipizide + metformin, 62.9 M/37.1 F HbA1c (%): Sitagliptin + metformin, 7.3; glipizide + metformin, 7.3 BMI (kg/m^2^): Sitagliptin + metformin, 30.9; glipizide + metformin, 31.3 Ethnicity: Caucasian, Black, Hispanic, Asian, other T2DM duration (years): 6.2–6.5	104	Primary: HbA1c level Other: FPG level, insulin level, proinsulin level, lipid profiles, β-cell function, (HOMA-β value) PR/IR, HOMA-IR quantitative insulin percentage with HbA1c <7%, incidence of adverse events, safety
Li, 2012, China (19)	Sitagliptin 100 mg qd + metformin ≥1500 mg qd Glimepiride 1–4 mg qd + metformin ≥1500 mg qd	N=116 Sitagliptin + metformin, n=58; glimepiride + metformin, n=58 Mean age (years): Sitagliptin + metformin, 53.7; glimepiride + metformin, 54.0 Sex ratio (%): Sitagliptin + metformin, 48.3 M/50 F; glimepiride + metformin, 50 M/50 F HbA1c (%): Sitagliptin + metformin, 8.8; glimepiride + metformin, 8.6 BMI (kg/m^2^): Sitagliptin + metformin, 26.7; glimepiride + metformin, 26.5 Ethnicity: Chinese Diabetes duration: NR	24	Primary: HbA1c level Other: percentage with HbA1c <7%, FPG level, 2HPPG level, incidence of adverse events
Koren, 2012, Israel (15)	Sitagliptin 100 mg qd + metformin ≥1500 mg qd Glibenclamide 5 mg qd + metformin ≥1500 mg qd	All patients: N=40 Mean age (years): 59 Gender ratio (%): 62.5 M/37.5 F HbA1c: 8.3% BMI (kg/m^2^): 31 Ethnicity: NR Diabetes duration: NR	12	Primary: arterial stiffness Other: HbA1c level, FPG level, blood pressure, lipid profiles, hsCRP level, BMI, STAT-8-isoprostane level, incidence of adverse events
Nauck, 2007, USA (16)	Sitagliptin 100 mg qd + metformin ≥1500 mg qd Glipizide 5–20 mg qd + metformin ≥1500 mg qd	N=1172 Sitagliptin + metformin, n=588; glipizide + metformin, n=584 Mean age (years): Sitagliptin + metformin, 56.8; glipizide + metformin, 56.6 Sex ratio (%): Sitagliptin + metformin, 57.1 M/42.9 F; glipizide + metformin, 61.3 M/38.7 F HbA1c (%): Sitagliptin + metformin, 7.7; glipizide + metformin, 7.6 BMI (kg/m^2^): Sitagliptin + metformin, 31.2; glipizide + metformin, 31.3 Ethnicity: Caucasian, Black, Hispanic, Asian, other Diabetes duration (years): 6.2–6.5	52	Primary: HbA1c level Other: FPG level, insulin level, percentage with HbA1c <7.0%, proinsulin lipid profiles, β-cell function (HOMA-β value, PI/IR HOMA-IR value, quantitative insulin index (QUICKI), incidence of adverse events, safety
Arechavaleta, 2011, USA (14)	Sitagliptin 100 mg qd + metformin ≥1500 mg qd Glimepiride 1–6 mg qd + metformin ≥1500 mg qd	N=1035 Sitagliptin + metformin, n=516; glimepiride + metformin, n=519 Mean age (years): Sitagliptin + metformin, 56.3; glimepiride + metformin, 56.2 Sex ratio (%): Sitagliptin + metformin, 55.0 M/46.2 F; glimepiride + metformin, 53.8 M/46.2 F HbA1c (%): Sitagliptin + metformin, 7.5; glimepiride + metformin, 7.5 BMI (kg/m^2^): Sitagliptin + metformin, 29.7; glimepiride + metformin, 30.2 Ethnicity: 37.8–38.0% Hispanic or Latino, 62–62.2% other Diabetes duration (years): 6.8–6.7	30	Primary: HbA1c level Other: FPG level, percentages with HbA1c <7.0 and <6.5%, lipid profiles, incidence of adverse events, safety
Srivastava, 2012, India (18)	Sitagliptin 50/100 mg qd + metformin ≥1500 mg qd Glimepiride 0.5 mg qd + metformin ≥1500 mg qd	N=50 sitagliptin + metformin, n=25; glimepiride + metformin, n=25 Mean age (years): NR Gender ratio (%): NR HbA1c (%): Sitagliptin + metformin, 8.28; glimepiride + metformin, 8.25 BMI (kg/m^2^): Sitagliptin + metformin, 25.27; glimepiride + metformin, 26.48 Ethnicity: NR Diabetes duration: NR	18	Primary: HbA1c level Other: percentage with HbA1c <7%, FPG level, 2HPPG level, BMI, incidence of adverse events

qd, once daily; HbA1c, glycated hemoglobin; BMI, body mass index; FPG, fasting plasma glucose; PPG, postprandial glucose; HOMA-IR, homeostasis model assessment of insulin resistance; NR, not reported; PI/IR, proinsulin/insulin ratio; 2HPPG, 2-h postprandial glucose; hsCRP, high-sensitivity C-reactive protein; T2DM, type 2 diabetes mellitus; M, male; F, female; QUICKI, quantitative insulin sensitivity check.

**Table II:** Results of quality assessment of six randomized controlled trials.

First author, year (ref)	Allocation concealment	Blinding	Randomization	Percentage that completed the trial	Intention- to-treat analysis	Free of selective reporting	Groups comparable at baseline
Nauck, 2007 (16)	Yes	Yes, double blind	Computer-generated allocation schedule	68	Yes	Yes	Yes
Arechavaleta, 2011 (14)	Yes	Yes, double blind	Computer-generated allocation schedule	90	Yes	Yes	Yes
Srivastava, 2012 (18)	Unclear	Unclear	Computer-generated random number	100	No	Yes	Yes
Seck, 2010 (17)	Yes	Yes, double blind	Computer-generated allocation schedule	43	Yes	Yes	Yes
Li, 2012 (20)	Unclear	Unclear	Random number table	100	No	Yes	Yes
Koren, 2012 (19)	No	Open-label crossover trial	Recruitment order	85	Yes	Yes	Yes
